# Wanted—A First-Class Mechanical Dentist

**Published:** 1887-04

**Authors:** Frank B. Darby


### "WANTED—A FIRST CLASS MECHANICAL DENTIST. NO STUDENT OR OPERATOR NEED APPLY."

BY FRANK B. DARBY D. D. S.

READ BEFORE A UNION MEETING OF THE 5TH, 6TH, 7TH AND 8TH DISTRICT DENTAL SOCIETY, HELD IN ROCHESTER, N. Y. OCT. 26 AND 27, 1886.

The above notice appeared in one of our journals not long since, and was responded to by twenty-three applicants, each claiming to be what the advertisement called for.

Six out of the twenty-three letters were well written and business-like communications; two addressed the advertiser as Mr., and signed themselves Doctor; five were so poorly written and spelled that they were consigned to the waste basket without answers. Still they earnestly solicited the "job," knowing they could "fill the bill." Two others were frank enough to acknowledge they knew all about laboratory work, "all kinds of metal work excepted." The remainder were of a school-boy composition kind, which suggested commendable labor and perseverance.

The time served by the several applicants ranged from three months to thirty years. One who had served a studentship of a few months, claimed to have had extraordinary facilities and wonderful perception, and had acquired the "whole thing" in that short time, but upon investigation had only mastered (in his own mind) red rubber. So one by one they dropped out of line as they were confronted with metal work, until but three remained.

This experience caused the writer to stop and inquire: What are we going to do?

The time has arrived when there is a growing demand for good laboratory men. Every dentist in full practice knows the importance of a first class mechanical assistant and knows also the difficulty in obtaining one. Taking a student for a term of two or three years means nothing, for as soon as he has acquired sufficient skill to render his service of value he is off to college, and soon into practice for himself.

The demand for skilled workmen has received a new impetus since the development in crown and bridge work, and as its possibilities are undoubtedly beyond our anticipations, we know not to what extent this demand may be carried, and as there is no limit to the inventive skill of our profession the busy operator must have some one at hand to work out and perfect his brain labor. The question of simply skilled mechanical labor in dentistry has been overlooked; in fact, the doors of the profession have been practically closed to those who might choose to adopt that branch as a life work. Encouragement has been given only to those who possess sufficient time, ability and means to perfect themselves for general practice, and those who have earned a diploma will rarely be found serving in the capacity of assistant.

It seems to me there is a place in dentistry for the mechanic; perhaps not in the broadest sense of the term, and surely not for the "rough and ready" man who knows nothing but work; but for the young man with a common school education, who by force of circumstances or chöice must learn a trade, and whose tastes are for light skilled labor, dental mechanics offers an inviting field. The steady, muscular individual, would undoubtedly make a better blacksmith or carpenter, while the dental laboratory would only be a congenial place for one more delicately organized.

If the demand is such that it is necessary to admit the apprentice into the sacred precincts of the laboratory, we can offer quite as great inducements as other occupations. The ordinary mechanic passes through an apprenticeship of three or four years, usually the former, receiving the first year $1.50 per week, second year $3.00, and the third year $5.00 per week. After serving his time the salary is raised according to the degree of skill acquired, rarely ever getting above $18.00 per week. All this we can offer, and more. We can promise light labor, short hours, genteel occupation, and if the dental office is what it should be, refined influences. We can place the young man in a better position socially; we can give him more hours for study if he wishes to enlarge his fund of general knowledge; he can have more time for recreation; in fact we can give him an honorable and respectable occupation in which he can command quite as much salary as in any trade, under like conditions.

Of course many a mechanic grows out of his journeymanship, out of his occupation in fact, by perseverance and development. In the same manner we would occasionally lose a valuable laboratory man, but the majority doubtless would be content. The idea of bringing the dental laboratory down to a competition with the trades may be looked upon by some as unprofessional, and the thought may not be practical, but the writer sees no way to meet the emergency unless the laboratories are thrown open, and young men given a chance. To do this practically there must be a thorough understanding that nothing but mechanical dentistry will be taught; no more study need be required than in any ordinary trade.

Dentists generally look upon students unfavorably, and are disposed to give the colleges the benefit of all applicants. Many a dentist has cause to repent in sackcloth and ashes the conscientious instructions given, when in later years he sees a man stepping into his shoes and reaping the benefit of an acquaintance made with his patients during studentship. Fortunately, the time has passed when a student can step out of the laboratory and hang up the sign of a dentist. The mountain-like diploma stands between him and success, and the young man who enters the profession now does so with the intention of taking a place in its ranks, and ere long there will be only temporary assistants who are preparing for college, or who labor between college courses.

Such an idea as this promulgated a few years ago (before the passing of the laws regulating practice), would have been disastrous; it would have opened the doors of respectability to every would-be mountebank, and placed the apprentice on an equal footing with the student. The time is not far distant when all colleges will be obliged to increase their facilities for instructions in the mechanical department, and it would not be a foolish prediction to declare that the time will come when they will teach this as a separate branch, and prepare men for skilled laboratory work alone.

In looking over a practice of twenty years, I am forced to believe that dentistry is just passing the border lines of a second development, which in time will astonish the world. The demands upon us are daily increasing, and the time will come when every man in full practice will find it impossible to accomplish his daily task without the helping hand of a skilled mechanic. It requires but a glance into the future to see red rubber, and other cheap dentures diminishing, and in the onward march of intelligence, metal work will again take its proper place and require of us a full surrender.

Every old root which in years passed was consigned to the merciless grip of the forcep, now invites our utmost skill, and the handiwork of a mechanic, and day by day we will find the burden heavier, and finally the overwhelming conviction will come that there are no skilled helpers.

